# Sustainable AFM-Based Nanolithography on Chitosan Thin Films for 2.5D and 3D Nanostructure Fabrication

**DOI:** 10.3390/nano16120724

**Published:** 2026-06-11

**Authors:** Lorenzo Vincenti, Isabella Farella, Mariafrancesca Cascione, Valeria De Matteis, Adriana Campa, Annalisa Bianco, Maurizio Martino, Fabio Quaranta, Alessandro Paolo Bramanti, Rosaria Rinaldi, Paolo Pellegrino

**Affiliations:** 1 Consorzio Interuniversitario Nazionale per la Scienza e Tecnologia dei Materiali (INSTM), V. Giuseppe Giusti, 9, 50121 Firenze, Italy; lorenzo.vincenti@unisalento.it; 2 Institute for Microelectronics and Microsystems (IMM), CNR, Via Monteroni, 73100 Lecce, Italy; isabella.farella@cnr.it (I.F.); adriana.campa@cnr.it (A.C.); fabio.quaranta@cnr.it (F.Q.); ross.rinaldi@unisalento.it (R.R.); 3 Department of Mathematics and Physics “E. De Giorgi”, University of Salento, Via Monteroni, 73100 Lecce, Italy; mariafrancesca.cascione@unisalento.it (M.C.); maurizio.martino@unisalento.it (M.M.); 4 Department of Experimental Medicine, University of Salento, Via Monteroni, 73100 Lecce, Italy; valeria.dematteis@unisalento.it (V.D.M.); annalisa.bianco@unisalento.it (A.B.); 5 STMicroelectronics S.r.l., System Research and Applications (SRA) Silicon Biotech, Via Monteroni, 73100 Lecce, Italy; alessandro.bramanti@st.com; 6 Scuola Superiore ISUFI, University of Salento, Via Monteroni, 73100 Lecce, Italy

**Keywords:** bio-resist, Atomic Force Microscopy, Atomic Force Microscopy-based nanolithography, nanostructures, nanolithography, sustainable materials, chitosan, biopolymer, eco-sustainability

## Abstract

The growing request for more sustainable materials and environmentally friendly nanofabrication methods in the electronics field has recently driven the scientific community in the development of bio-derived materials as an alternative to conventional lithographic resists. In this work, we used chitosan, a biodegradable and biocompatible polysaccharide, as a green direct-write resist material for Atomic Force Microscopy-based nanolithography. Chitosan thin layers were obtained by spin coating and systematically characterized, in terms of thickness and surface roughness, demonstrating nanoscale smoothness and tunable film thickness. Three Pulse–Atomic Force Lithography (P-AFL) approaches, i.e., Constant Pulse, Gradient Pulse, and Raster Pulse AFL methods, were used to pattern nanostructures with constant-depth nanogrooves, variable-depth (2.5D) profile, and three-dimensional nanoholes on chitosan films. The results reveal high pattern fidelity, reproducibility, and tunability of feature dimensions as a function of applied force and scanning direction. Moreover, the RP-AFL technique enabled the fabrication of well-defined 3D nanostructures with depths matching the film thickness, which is a prerequisite for subsequent pattern transfer. This experimental work provided a first proof-of-concept to adopt chitosan as a more sustainable alternative with respect to conventional resists. Moreover, the results highlight P-AFL methods as a versatile and low-impact nanofabrication strategy, contributing to the development of greener micro- and nano-manufacturing technologies.

## 1. Introduction

The electronics industry is a continuously growing sector, where technological innovations are primarily evaluated in terms of the efficiency and performance of new devices, thereby shaping trends in the global electronics market [1,2,3]. While many resources are invested in optimizing performance and sourcing raw materials at affordable prices, attention to environmental drawbacks is rising, shifting the focus to the importance of non-harmful chemicals and eco-sustainable practices [4,5,6,7].

In addition to the environmental aspects, the ongoing global semiconductor chip shortage since 2020 demonstrates that the raw materials supply chain, which supports the entire electronics industry, is exposed to geopolitical tensions and conflicts, global dynamics (such as the COVID-19 pandemic), and the exploitation of natural resources [8]. The growing demand for electronic devices has increased resource consumption, while their relatively short lifetimes have contributed to worsening the global electronic waste (e-waste) crisis of the last few decades [9,10]. In this framework, according to the Global E-waste Monitor, more than 53.6 million tons of e-waste were produced worldwide in 2020, and this situation does not seem likely to improve in the coming years: indeed, is projected to increase to 82 million metric tons by 2030 [11,12]. A large amount of this waste is made up of non-biodegradable and potentially dangerous materials, such as heavy metals and brominated compounds, that can seep into the environment and harm ecosystems, as well as human health [13,14]. To try to address this problem, many recycling-based strategies have been developed. Anyway, current recycling methods are still far from ideal: those methods tend to be inefficient and energy-demanding, often focusing only on recovering valuable metals while overlooking the wider environmental impact [15,16].

In the last decade, this shift has been further stimulated by the high-tech industrial guidelines and regulatory frameworks, with the aim of reducing the environmental impact of electronic device fabrication processes. More in detail, a great interest is focused on the development of innovative and even more efficient strategies towards more sustainable micro- and nanofabrication methods, which are fundamental in modern electronics, without the massive use of hazardous chemicals and non-renewable materials [17]. Unfortunately, the fabrication of all those devices typically involves polymeric resists, organic solvents, and toxic developers, which have a tremendous impact on both human health and the ecosystem [18,19]. Developers such as tetramethylammonium hydroxide (TMAH) present severe toxicity risks [20,21], while conventional resists, e.g., poly(methyl methacrylate) (PMMA), depend on petroleum-derived feedstocks and solvent-intensive processing [22]. Collectively, these factors contribute to environmental pollution, safety challenges, and increased disposal complexity [23].

In this framework, the efforts of the scientific community have been directed toward the development of both green materials for lithography and nanofabrication [3]. Bio-derived, biodegradable, and biocompatible polymers have emerged as particularly attractive candidates due to their renewability, low toxicity, and functional versatility, permitting them to be integrated into high-tech devices [2]. Bio-derived materials such as cellulose, silk fibroin, chitin, chitosan, galactomannans, and natural resins have a combination of mechanical robustness and environmentally benign processing [24,25].

Indeed, recent studies have demonstrated that such materials can be effectively integrated into lithographic workflows, enabling “green” nanofabrication approaches based on water-processable or solvent-free systems [17,26]. High-resolution features have been achieved using silk-based and saccharide-based resists, in some cases reaching sub-50 nm dimensions with successful pattern transfer [25,27]. However, many of these strategies still rely on additional processing steps, such as chemical functionalization to enhance sensitivity or the use of multilayer stacks incorporating synthetic polymers. These requirements partially offset the environmental benefits by introducing added chemical complexity and non-renewable components [18,25]. At the same time, due to the urgency of transitioning to sustainable materials and processes, the scientific community has responded by increasing research activity in the field, as evidenced by the number of publications on eco-friendly electronic materials, which grew nearly twentyfold between 2010 and 2020 [28].

In addition, innovative and alternative nanofabrication techniques are also being explored in order to reduce energy consumption and the use of hazardous chemicals. Innovative patterning approaches such as nanoimprint lithography offer high-resolution patterning with an easy processing approach and reduced reliance on hazardous chemicals [29,30,31]. In the meantime, conventional techniques, including photolithography [32], Edge lithography [33], electron-beam lithography [34,35], Inkjet printing [36], Phase-Shift Lithography [37,38,39,40], AFM-Based Nanolithography [41], Dip-pen nanolithography [42], DUV lithography, and laser writing [43] are explored for compatibility with bio-based materials [25,36,37,38,44,45]. Moreover, the bottom-up strategies, such as block copolymer self-assembly [46,47,48] and DNA-based nanofabrication, further expand the available toolbox by exploiting intrinsic molecular organization [49,50,51]. The aforementioned green nanofabrication methods offer a more sustainable alternative to conventional lithographic processes. These approaches generally consume less energy and generate lower amounts of waste, helping to decrease the environmental impact of nanoscale manufacturing [17]. In addition, the use of biodegradable and bio-derived materials, combined with low-impact techniques such as AFM-based nanolithography, opens new possibilities for the fabrication of environmentally friendly electronic and biomedical devices without compromising nanoscale precision [43].

Within this context, this work focuses on the use of chitosan, a bio-derived polysaccharide obtained from chitin, as a sustainable direct-write resist material for AFM-based nanolithography [41,52]. Chitosan combines several advantageous properties, such as biodegradability, biocompatibility, film-forming capability, and compatibility with aqueous processing [53,54]. Unlike many previously reported bio-based resists, this approach does not require chemical functionalization or complex multilayer architectures, thereby simplifying fabrication while preserving environmental benefits. Furthermore, chitosan can function not only as a resist but also as an effective mask for pattern transfer, providing a streamlined and eco-friendly route toward high-resolution nanofabrication.

In this paper, we carried out the realization of a smooth and homogeneous chitosan film by optimizing the synthesis of proper solutions (with a polymer concentration ranging from 0.7% to 0.9% *w*/*v*%) and their subsequent spin coating onto a clean silicon dioxide substrate. After their characterization, in terms of sample surface roughness and thickness, the 0.7% chitosan layer was selected and used as a substrate for the patterning of several nanostructures. More in detail, the proposed nanofabrication processes successfully combine chitosan with unconventional AFM-based nanolithography techniques. Indeed, Constant Pulse (CP-), Gradient Pulse- (GP-), and Raster Pulse- (RP-)Atomic Force Lithography (AFL) were adopted to realize 1D, 2.5D, and 3D nanostructures on chitosan thin films, respectively. These strategies are aligned with the principles of green nanotechnology, as the absence of toxic photoresists and developers, together with the use of a natural and biodegradable polymer, aims to reduce the environmental impact of the life-cycle of electronic devices from production to disposal. This study represents a proof-of-concept demonstrating that a biopolymer, chitosan, can be processed sustainably into homogeneous films on a hard substrate and coupled with a non-conventional nanolithography technique, that is P-AFL, to manufacture a variety of different nanostructures.

## 2. Materials and Methods

### 2.1. Chitosan Solution Preparation

**Figure 1:**
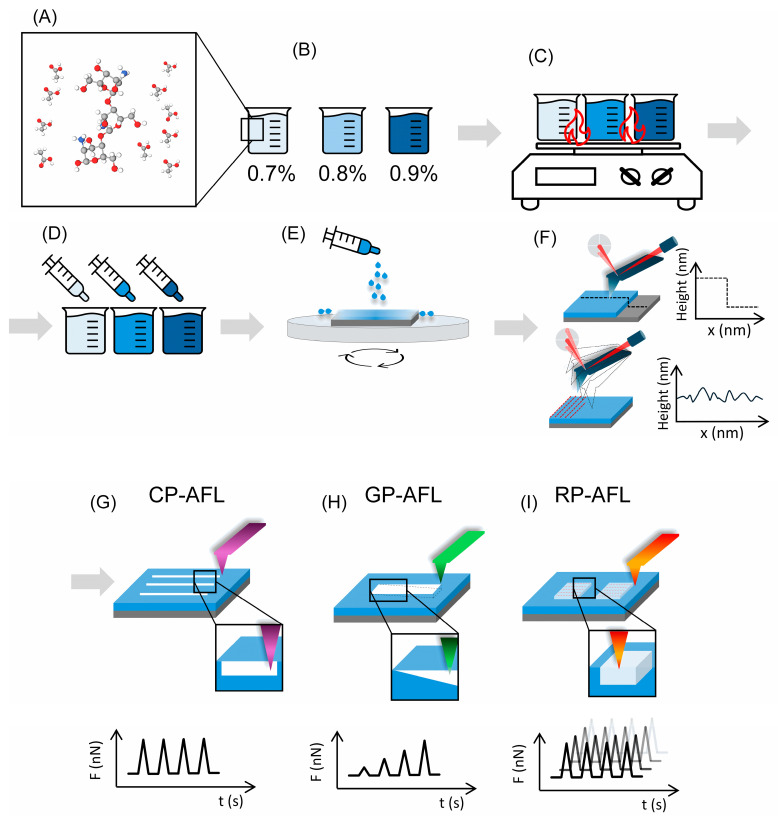
Schematic representation of the nanolithography experiments process flow: (**A**) chitosan molecules dissolved in Acetic Acid Solution (Red Dots: Oxygen; Grey Dots: Carbon, White Dots: Hydrogen; Blue Dots: Nitrogen); (**B**) Ch solutions at 0.7%, 0.8%, and 0.9% *w*/*v*. (**C**) Ch solutions were heated to 80 °C under stirring at ~800 rpm in order to dissolve the polymer particles; (**D**) polymer solutions were further filtered to remove undissolved Ch powder. (**E**) The Ch filtered solutions were spun on clean SiO_x_ substrates and (**F**) subsequently imaged by AFM in order to quantify the film thickness and surface roughness. The Ch films were then used as substrates for the patterning of (**G**) nanogrooves with constant depth, (**H**) nanotrenches with varying depth, and (**I**) three-dimensional nanosquares by CP-AFL, GP-AFL, and RP-AFL, respectively.

The chitosan (Ch) powder with a medium molecular weight and a degree of deacetylation ≥ 75% was dissolved in an aqueous 1% *v*/*v* acetic acid solution (Figure 1A). The polymer concentration in the solutions was equal to 0.7%, 0.8%, and 0.9% *w*/*v* (Figure 1B). To ensure complete polymer powder dissolution, the chitosan solutions were heated to approximately 80 °C and stirred continuously at approximately 800 rpm for about 8–12 h (Figure 1C). Afterward, the solutions were filtered using cellulose acetate syringe filters with pore sizes of 0.45 μm and 0.22 μm, applied sequentially to remove any remaining undissolved particles or impurities (Figure 1D). Chitosan, glacial acetic acid (≥99.7% purity), and syringe filters were purchased from Sigma-Aldrich (Merck KGaA) (Darmstadt, Germany).

### 2.2. Substrate Preparation

A 3-inch silicon wafer coated with a 0.5 µm thermally grown silicon dioxide layer (p-type, boron-doped, ⟨100⟩ orientation, resistivity 1–30 Ω·cm; Si-Mat Silicon Materials, Kaufering, Germany) was used as the substrate. Before use, the wafer was cleaned by sequential immersion in heated ethanol and 2-propanol baths, each for at least 15 min. Successively, the sample was rinsed with abundant deionized water and dried under a nitrogen stream for about 60 s. To further improve surface cleanliness, the substrates were treated in a piranha solution (3:1 *v*/*v* mixture of H_2_SO_4_ and H_2_O_2_) for 60 min. After this step, they were thoroughly rinsed with deionized water three times and dried out with a nitrogen stream for 30–60 s. In order to desorb any residues of H_2_O, samples were baked at 150 °C for 10 min on a hotplate. The cleaned wafers were then cut into smaller sections, yielding 2 cm × 2 cm chips. Chitosan solutions (prepared in 1% *v*/*v* acetic acid and kept at an ambient temperature of ~20 °C) were deposited onto the SiO_2_ substrates. In more detail, 250 µL of chitosan solutions were deposited onto the silicon substrate fixed on the spin coater sample holder, subsequently the Ch solutions were rested on the surface for 3 min before spin coating at 34 rps for 120 s (SCC-200 spin coater, Schaefer Technologie GmbH, Langen, Germany) (Figure 1E). Finally, the coated substrates were heated at 70 °C for 5 min to remove residual solvent and promote film stabilization. The surface morphology and thickness of the resulting chitosan films were characterized by Atomic Force Microscopy (AFM) in semicontact mode.

Ethanol (99.5%), 2-propanol (99.5%), hydrogen peroxide (30%), and sulfuric acid (95–98%) were also obtained from Sigma-Aldrich (Merck KGaA) (Darmstadt, Germany).

### 2.3. Instrumentation for AFM Measurements and Pulse–Atomic Force Nanolithography

The morphology of the chitosan substrates and the AFM-patterned nanostructures was investigated using a commercial AFM system (NTEGRA, NT-MDT Spectrum Instruments, Moscow, Russia), equipped with both spectroscopy and nanolithography modules. All measurements were carried out under ambient laboratory conditions (approximately 20 °C and 60% relative humidity).

The High-resolution topographic imaging was performed by using NSG01 probes (NT-MDT Spectrum Instruments), characterized by a nominal tip radius of ~6 nm, a resonance frequency of ~150 kHz, and a spring constant equal to approximately 5.1 N/m. Images were acquired in semicontact error mode with a setpoint of ~5.0 nA, a feedback gain of 0.80, and a scan rate of 0.5 Hz. In semicontact error mode, two signal channels were simultaneously recorded: the SensHeight channel, providing quantitative topographic information, and the Magnitude (Mag) channel, which reflects the amplitude error signal of the feedback loop. Therefore, the Mag signal captures deviations of the cantilever oscillation amplitude from the setpoint. To correct for background artifacts such as sample tilt and bow, a second-order surface subtraction was applied to the raw SensHeight data. Surface roughness was calculated as the root-mean-square (R_q_) of the height distribution over 10 areas 2 µm × 2 µm, randomly selected from each sample, according to Equation (1):

(1) $$R_{q}=\sqrt{\frac{1}{n}\sum_{i=1}^{n}z_{i}^{2}}$$

where *n* represents the total number of data points (256 × 256 in this study), and *z_i_* is the deviation of each point from the mean surface height [55,56]. The reported *R_q_* values are expressed as mean ± standard deviation (SD), obtained from five independent areas per sample.

The P-AFL experiments were performed using commercial Au-coated NSG30 probes (NT-MDT Spectrum Instruments) equipped with single-crystal silicon tips (n-type, antimony-doped, resistivity 0.01–0.25 Ω·cm). The probes consist of a rectangular cantilever with a nominal resonance frequency between 240 and 440 kHz (typically ~320 kHz) and a tetrahedral apex supporting a conical silicon tip with a nominal radius of approximately 30 nm. The nominal spring constant of the cantilever, as specified by the manufacturer, ranges from 22 to 100 N/m.

### 2.4. AFM Nanolithography Method

The nanostructures reported in this paper were patterned onto chitosan thin films via AFM-based nanofabrication techniques. In this work, three different AFM-based nanofabrication methods were employed: Constant Pulse–Atomic Force Lithography (CP-AFL), Gradient Pulse–Atomic Force Lithography (GP-AFL), and Raster Pulse–Atomic Force Lithography (RP-AFL). All these techniques, derived from the Pulse–Atomic Force Lithography (P-AFL) framework previously developed in our studies [57,58,59,60,61], enable precise mechanical nanostructuring of polymer surfaces via controlled nanoindentation.

Generally speaking, P-AFL works by modulating the interaction forces between the AFM tip and the sample to locally deform the material. First, the probe is brought into contact with the surface, and a symmetric triangular voltage pulse train of appropriate amplitude is then applied to the piezoelectric scanner. This produces a controlled vertical motion of the cantilever, allowing the tip to repeatedly and reliably penetrate the material, creating a well-defined nanostructure on the chosen substrate. Depending on the amplitude of the electric signal, either kept constant or varied from one indentation to the next one, the technique can be used in different ways to create both two-dimensional (2D) and quasi-three-dimensional (2.5D) structures. Moreover, the fabrication of fully three-dimensional (3D) structures can be achieved by moving the tip across the area to be patterned in raster mode, while maintaining a constant force and cantilever deflection.

It is worth reminding that the depth of each nanostructure is strongly influenced by the cantilever deflection setpoint, which is directly related to the applied normal force, but is further influenced by the probe geometry and the mechanical properties of the chitosan film. Continuous nanogrooves were generated through a sequence of closely spaced indentations (“Step”) along predefined paths. Based on prior optimization, the step size must not exceed half of the tip radius to ensure structural continuity. Accordingly, using probes with a nominal radius of 30 nm, a step size of 10 nm was selected, corresponding to the minimum lateral resolution of the piezo-scanner. The pulse width per indentation was fixed at 300 ms, while the lateral scan velocity was maintained at 0.25 µm/s.

To systematically investigate the effect of applied force on groove morphology and depth, the setpoint was varied from 0.5 to 6.0 nA in increments of 0.5 nA. The corresponding force values were calibrated via force–distance spectroscopy measurements. During lithography, pulse parameters were continuously monitored using an oscilloscope, and the AFM feedback loop ensured precise lateral positioning. Following fabrication, the resulting nanostructures were characterized by AFM to assess their morphological and geometrical features.

### 2.5. Force Spectroscopy

Because the P-AFL critically depends on the force applied by the AFM tip during indentation, force spectroscopy was first used to quantify these force values prior to performing the P-AFL experiments.

When the AFM probe is in contact with the sample surface, variations in the voltage applied to the z-section of the piezo-scanner produce vertical movements of the cantilever, which are detected as corresponding changes in the deflection (DFL) signal. By examining the relationship between DFL and Z and applying Hooke’s law (Equation (2)),

F = kΔz(2)

where F represents the applied force, k is the cantilever spring constant (N/m), and Δz is the vertical displacement (nm), the force applied on the AFM tip for each setpoint can be calculated. The cantilever stiffness was calibrated using the thermal noise method [62,63,64]. The force required to induce a permanent deformation or penetration of a material depends strongly on its mechanical properties, surface characteristics, and AFM tip properties. Soft materials, such as polymers, can often be patterned using forces in the nanonewton range, whereas harder materials may require significantly higher forces. Under the force conditions employed in this work, the silicon substrate used for calibration can be considered non-deformable [65,66]. In this paper, the force spectroscopy analysis was performed on 15 setpoint values ranging from 1.0 nA to 6.0 nA, with increments of 0.5 nA. These setpoints were then used consistently in all subsequent lithography experiments.

### 2.6. Software

AFM topographical analyses were performed using NOVA_PX software. Image processing, including correction of bow and three-dimensional distortions in AFM scans, as well as surface roughness quantification, was carried out using Image Analysis P9 (IA-P9). Both software tools were provided by NT-MDT Spectrum Instruments (Moscow, Russia).

The morphology of the nanogrooves was further analyzed using ImageJ (version 1.47v, National Institutes of Health, Bethesda, MD, USA). Experimental data were processed and visualized using OriginPro 2024b (OriginLab Corporation, Northampton, MA, USA). To improve accessibility and ensure accurate interpretation, all graphical outputs were generated using colorblind-friendly palettes [67,68]. The force spectroscopy analysis and the extrapolation of force parameters were performed by a custom-made, properly developed Python code.

### 2.7. Statistical Analysis

Statistical analysis was performed using one-way analysis of variance (ANOVA), followed by Tukey’s honestly significant difference (HSD) test for post hoc comparisons, exclusively to the thickness and roughness datasets, while all the other measurements are reported as mean ± standard deviation and were used to describe the reproducibility and trends of the nanofabrication process. A significance level of *p* ≤ 0.05 was considered statistically significant. Unless otherwise specified, all data are reported as mean ± standard deviation (SD), based on five independent replicates. In more detail, the notation *n* = *x* reported in the figure captions indicates the number of independently fabricated nanostructures produced under identical experimental conditions (*n* = 3 or 5, depending on the specific experiment).

## 3. Results and Discussion

### 3.1. Morphological Characterization of Chitosan Films by AFM

**Figure 2:**
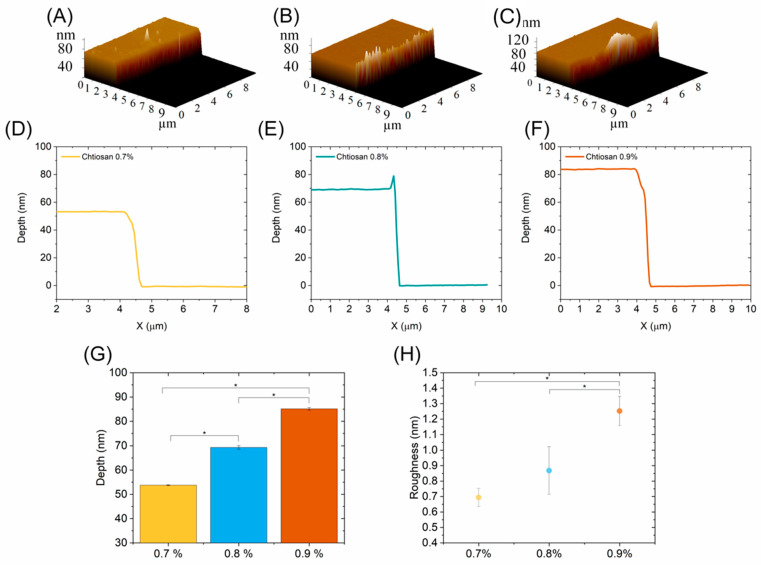
High-resolution 3D AFM images acquired in the SensHeight channel of chitosan layers at concentrations of (**A**) 0.7%, (**B**) 0.8%, and (**C**) 0.9% *w*/*v*%. Cross-sectional profiles, randomly acquired on the AFM images of (**D**) 0.7%, (**E**) 0.8%, and (**F**) 0.9% *w*/*v*% chitosan layers. (**G**) Mean Ch film thickness at concentrations from 0.7% to 0.9% *w*/*v*, in 0.1% increments, and (**H**) corresponding average roughness values (R_q_) estimated from ten randomly selected (2 × 2) μm^2^ areas on the AFM images. The data are reported as mean value ± SD; error bars represent standard deviation; statistical significance is denoted by * *p* ≤ 0.05.

Chitosan (Ch) thin films were fabricated by spin coating solutions prepared by dissolving Ch powder in 1% *v*/*v* acetic acid at a concentration of 0.7%, 0.8% and 0.9% *w*/*v*%, as described in the Section 2.1. The choice of using acetic acid is due to its safety, low cost, and effectiveness as a solvent. After the solution filtration, the Ch solutions were deposited by spin coating on clean Silicon substrates; then, the films were characterized using AFM in semicontact error mode with NSG01 probes. The 2D topographical images confirmed that the films were uniform, continuous, and free of visible defects, with good thickness homogeneity and strong adhesion to the substrate. The Ch layer thickness was measured by scratching the Ch film with a pipette plastic tip and, successively, the scratches were cleaned with a nitrogen stream for 60 s, in order to remove polymer debris. The scratches on chitosan layers were analyzed by AFM profiles for estimating the film thickness and plotted in Figure 2. A clear concentration-dependent trend was observed, with thickness increasing from (53.78 ± 0.19) nm at 0.7% *w*/*v* to (69.32 ± 0.74) nm at 0.8% *w*/*v*, and reaching (85.16 ± 0.54) nm at 0.9% *w*/*v*, demonstrating precise control over film thickness via spin coating.

Moreover, the surface morphology was characterized in terms of root-mean-square (R_q_) roughness analysis over multiple (5 × 5) µm^2^ areas. The measured R_q_ values were (0.69 ± 0.06) nm, (0.87 ± 0.15) nm, and (1.25 ± 0.09) nm for 0.7%, 0.8%, and 0.9% *w*/*v* Ch films, respectively. These results indicate a slight increase in the Ch surface roughness as the polymer concentration increases, while maintaining an overall nanoscale smoothness. Taking into account the thicknesses of the Ch film and their R_q_ values, the 0.7% chitosan films are compatible with the P-AFL test, as reported in our previous studies [57,58,59,60,61]. Indeed, the combination of a thickness less than 60 nm and a smooth surface (<1 nm) makes the 0.7% films suitable for nanoscale pattern transfer, supporting good resolution and limited feature distortion.

### 3.2. Pulse–Atomic Force Lithography

Before fabricating nanostructures by using the Pulse–Atomic Force Lithography technique, it was necessary to accurately determine the normal force applied by the AFM probe to the sample surface. As described in the Section 2.4, to measure the normal force established between the AFM probe and the substrate during the nanoindentations, force spectroscopy experiments were carried out in contact mode on a silicon wafer using an NSG30 AFM probe. The corresponding Force–Distance (FD) curves, reflecting how cantilever deflection changes with the vertical movement of the piezoelectric scanner, were collected over a setpoint range from 0.5 to 6.0 nA, with increments of 0.5 nA. For each setpoint value, ten FD curves were recorded using NOVA-PX software to ensure consistency in the measurements.

The collected data were then analyzed using a custom Python script to determine the average normal force and its standard deviation for each setpoint. The force values were calculated from the slope of the contact region of the FD curves, together with the cantilever spring constant, as described in Equation (2). The Force values were reported in Table 1.

Each setpoint value was used to create nanostructures by the P-AFL methods on Ch films prepared from chitosan solutions at a concentration of 0.7%, employing NSG30 probes.

#### 3.2.1. Constant Pulse–Atomic Force Lithography

A total of 12 nanogrooves with uniform depth and a length of 1 µm were fabricated by progressively increasing the setpoint. All other lithographic parameters (including pulse duration, scan velocity, and environmental conditions) were maintained constant to ensure that any variation in groove depth could be attributed solely to changes in the applied normal force.

**Figure 3:**
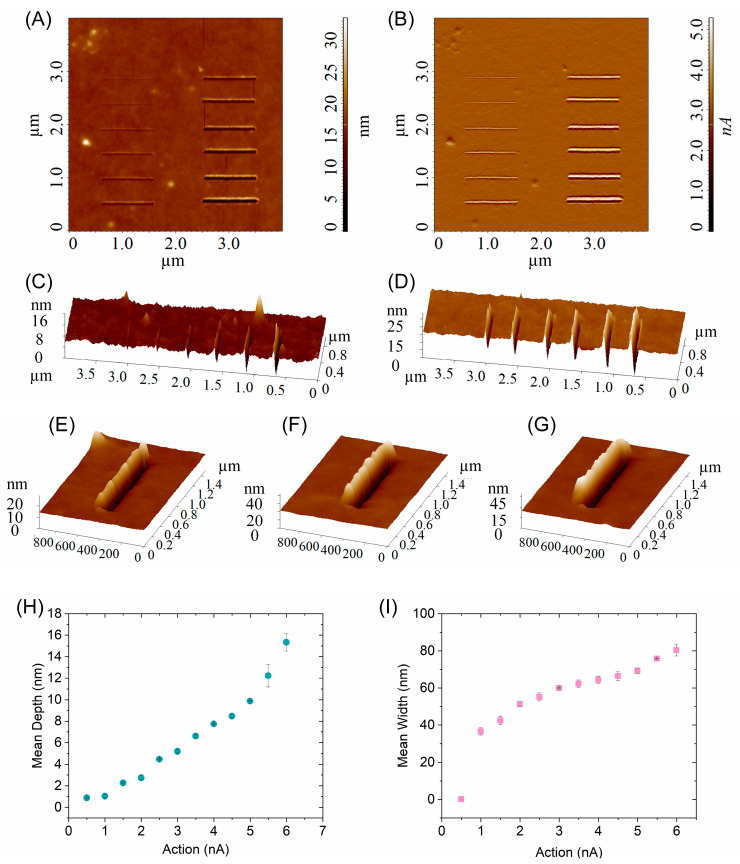
(**A**) 2D High-resolution AFM topography image of a constant-depth nanogrooves array on Ch films, obtained by spin coating of chitosan solutions at a concentration of 0.7% on SiOx substrate. The AFM SensHeight image is shown on the left, and (**B**) the Magnitude Error image on the right. (**C**,**D**) 3D images of the constant-depth nanogrooves, cropped in the middle of the nanochannels, to better observe the variation in nanostructure depth as a function of the applied force. (**E**–**G**) 3D AFM topographies of nanogrooves patterned with a setpoint equal to 3nA, 5nA, and 6nA, respectively. (**H**) Mean nanogroove depth and (**I**) mean width values, plotted as a function of the Setpoint. The data in the graphs were reported as mean value ± standard deviation (*n* = 3).

Subsequent topographical analysis of the patterned nanogroove arrays was performed using AFM in semicontact error mode with NSG01 probes, which possess a sharper tip radius compared to NSG30 probes. This imaging mode was selected to minimize further surface alteration while enabling high-resolution characterization. The AFM images presented in Figure 3 demonstrate that the fabricated nanogrooves closely match the intended design, exhibiting a high degree of order, uniform spacing, and consistent morphology across the patterned regions. Moreover, no appreciable distortions, defects, or irregularities were observed, indicating excellent reproducibility of the fabrication process. The groove morphology showed a clear dependence on the applied force: the increase in the setpoint (then, the force applied) led to a corresponding increase in both groove depth and width. Furthermore, the pile-up features, representing displaced polymer material accumulating along the groove edges, also increased in height with higher applied forces (Figure 3A–D).

At low setpoint values (below approximately 0.5–1.5 nA), the resulting nanostructures were extremely shallow and narrow, often appearing faint or poorly resolved in SensHeight images. This behavior can be attributed, at least in part, to the relatively soft nature of the chitosan substrate. In contrast, setpoint values exceeding 2.0 nA produced well-defined grooves with greater depth and width, which were clearly resolved in both SensHeight and Mag images. This finding is more evident by observing the three-dimensional AFM images, cropped on the middle part of the nanogrooves: the depth of the grooves increases as the setpoint increases, as well as the pile-ups surrounding the nanochannels.

More in detail, as the force applied increases, ranging from setpoint 0.5 nA (~0.2 µN) to setpoint 6 nA (~2.41 µN), the nanogrooves’ depth varied from (0.8 ± 0.1) nm to (15.3 ± 0.8), as reported in Figure 3C. Similarly, the increase in the normal force applied led to an increase in the nanochannel width, reaching a width vale equal to (80.3± 3.2) nm in the nanostructures patterned with the higher setpoint value (Figure 3D).

**Figure 4:**
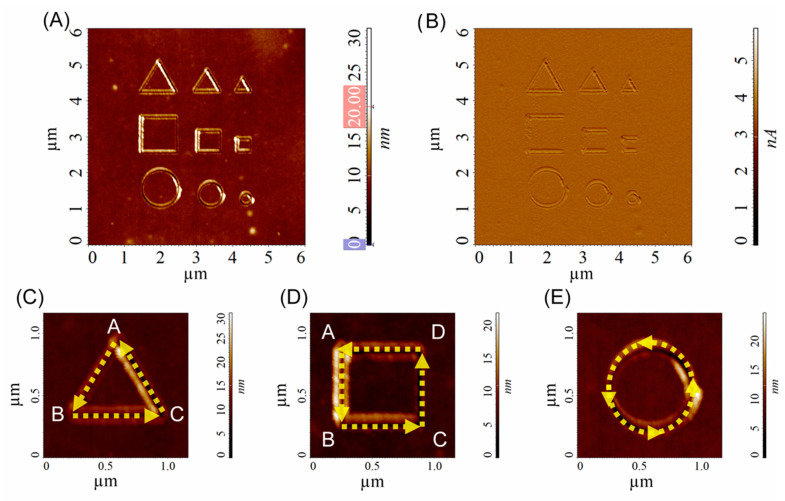
High-resolution AFM images of constant-depth nanostructures patterned on Ch films, including triangles, squares, and circles of varying sizes. (**A**) SensHeight (topography) image and (**B**) corresponding Magnitude Error image. Schematic representation of the nanopatterning paths used to fabricate (**C**) nanotriangles, (**D**) nanosquares, and (**E**) nanocircles, indicating the tip scanning direction during structuring (*n* = 3).

An additional parameter investigated in this paper was the patterning direction of the AFM tip during the lithographic process. Indeed, the NSG30 probes are characterized by a tetrahedral apex geometry and are expected to induce direction-dependent variations in nanostructure morphology due to anisotropic tip–sample contact mechanics and asymmetric stress distribution at the interface. To assess the performance of the CP-AFL technique by using NSG30 tips, complex nanogroove architectures were fabricated on 0.7% Ch films. More in detail, (i) square patterns were employed to probe lithographic behavior along orthogonal (horizontal and vertical) directions, (ii) triangular shape nanostructures were used to investigate oblique patterning, and (iii) circular features were chosen to evaluate the capability of the CP-AFL of generating continuously curved structures involving all scanning orientations on the Ch layers. All the patterns were fabricated at lateral dimensions of 800 nm, 600 nm, and 400 nm, corresponding to the side length of squares and triangles, and the diameter of circular features. All the other nanolithographic parameters were maintained constant to ensure experimental control: a setpoint of 6.0 nA, a pulse duration of 300 ms, a scan velocity of about 0.25 μm/s, a triangular voltage, and a lateral step size of 10 nm between successive indentations. After the nanostructures’ fabrication, characterization was performed via high-resolution AFM imaging in semicontact mode using NSG01 probes, whose reduced tip radius ensures improved spatial resolution while minimizing additional surface perturbation. The SensHeight and Magnitude images (Figure 4) show that all patterned nanostructures exhibit an overall well-defined shape, high pattern fidelity, and the grooves’ cavity appears free of polymer debris inside. Anyway, the displaced polymer accumulates predominantly at the groove edges, forming pile-up features all around the tranches.

Despite the overall uniformity of the fabricated structures, a pronounced anisotropy in groove morphology as a function of scanning direction is observed. In particular, the nanogrooves generated in the bottom-to-top direction exhibit larger lateral dimensions compared to those patterned in the opposite direction, whereas comparable widths are observed along vertically oriented segments. Significant variations are also evident in groove depth.

To better characterize this observation, a quantitative morphometric analysis was carried out by extracting six independent cross-sectional profiles for each side of the 800 nm square, following the nomenclature defined in Figure 4. The resulting values indicate the following: A→B: depth = (31 ± 2) nm, width = (68 ± 3) nm; B→C: depth = (21 ± 1) nm, width = (62 ± 2) nm; C→D: depth = (11.5 ± 0.3) nm, width = (62 ± 2) nm; D→A: depth = (20.8 ± 0.3) nm, width = (63 ± 2) nm.

The most pronounced deviation is observed for the C→D segment, whose depth is approximately two to three times smaller than those measured for the other sides of the square pattern. This behavior can be rationalized in terms of direction-dependent cantilever deflection and tip bending mechanisms. Specifically, during bottom-to-top scanning (i.e., with the cantilever advancing toward the patterned region), the effective loading conditions at the tip–sample interface may differ substantially from those occurring in the reverse direction, leading to a reduced indentation efficiency and consequently shallower grooves. Conversely, in horizontal scanning configurations, the bending dynamics of the cantilever are expected to remain invariant, resulting in more consistent feature dimensions.

#### 3.2.2. Gradient Pulse–Atomic Force Lithography

**Figure 5:**
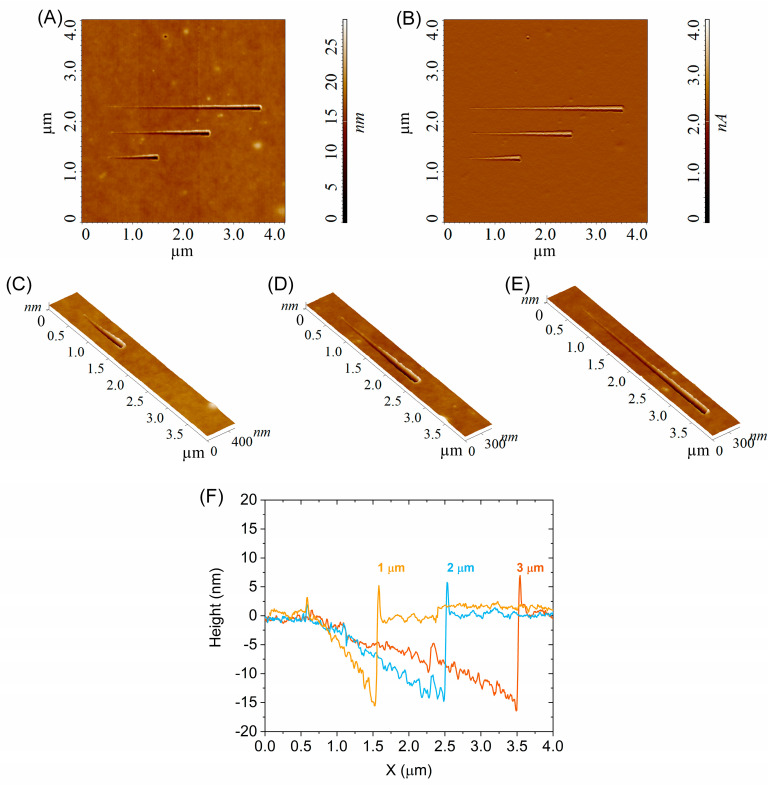
AFM topography images of varying depth nanostructures, with a length of 1, 2, and 3 µm, patterned on chitosan film by GP-AFL, acquired on (**A**) SensHeight and (**B**) Mag channels. Topographic 3D AFM images of nanogrooves with a length of (**C**) 1 µm, (**D**) 2 µm, and (**E**) 3 µm. (**F**) Cross-section of the nanotranches by which it is possible to appreciate the varying-depth profile of the varying-depth patterned nanogrooves (*n* = 3).

In the GP-AFL, two nanopatterning parameters are fundamental: Action1 (nA), corresponding to the initial setpoint value, and Action2 (nA), which corresponds to the final setpoint value used in the nanolithography process. Every single indentation along the predefined patterns is carried out with an action value (then, a proper force) that varies linearly between Action1 and Action2. As previously reported, the nanogrooves with graded depth were nanofabricated on a Ch layer obtained from a 0.7% polymer solution, using an AFM NTEGRA system equipped with NSG30 probes. All the nanostructures were produced under the same patterning conditions, i.e., Action1 equal to 0.0 nA, Action2 values of 6.0 nA (corresponding to a force value of about (2.41 ± 0.08) µN), a triangular (symmetric) voltage pulse with a duration τ = 300 ms, scan speed of 0.25 μm/s, and step = 10 nm (spacing between two following indentations). The varying-depth nanogrooves were patterned within an area of approximately 4 × 4 μm^2^, with lengths of 1, 2, and 3 μm. After patterning, these 2.5D nanostructures were imaged in semicontact mode by means of NSG01 tips. After applying a first-order flattening filter to the SensHeight data, the resulting image is presented in Figure 5A, while the corresponding Mag image is shown in Figure 5B. To better appreciate the nanochannel shape, three-dimensional images of a varying-depth nanochannel are reported in Figure 5C–E. The cross-section profiles, acquired on the inner part of the nanogrooves and representing the depth variation along the grooves, were acquired by using IA-P9 software, and then plotted with Origin2018, as shown in Figure 5F.

As shown in Figure 5, the fabricated nanostructures exhibit high uniformity and clearly defined morphology. Moreover, as observed from the cross-sections, the nanostructures’ depth profiles range from the baseline level of the pristine Ch surface (ideally equal to 0.0 nm) to a maximum depth governed by the Action2 parameter, in agreement with the calibration data. The depth variation along the nanogrooves follows an almost linear trend, except in the portions patterned with lower action values, corresponding to the lower force values reported in Table 1. Moreover, the measured profiles are consistent with those obtained for constant-depth nanogrooves discussed in the previous section, termed “Constant Pulse–Atomic Force Lithography”. These results also confirm not only the reproducibility of the GP-AFL method and the transferability of calibration from CP-AFL, but also its capability for precise control in the fabrication of variable-depth (2.5D) nanostructures on biopolymer substrates.

#### 3.2.3. Raster Pulse–Atomic Force Lithography

**Figure 6:**
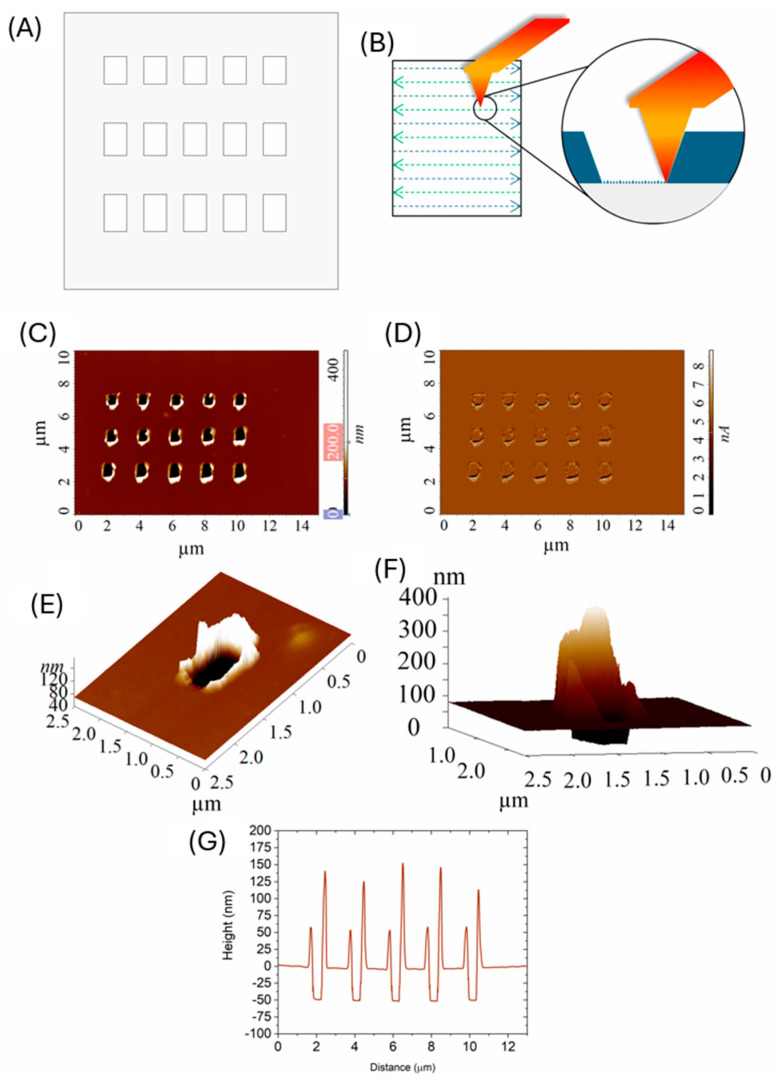
(**A**) RP-AFL nanopatterning template of the 3D nanoholes and (**B**) sketch representation of the AFM tips indentations in RP-AFL. AFM image of the 3D nanohole array patterned on the chitosan layer acquired in the (**C**) SensHeight and (**D**) Mag channels, respectively. (**E**,**F**) Three-dimensional AFM images of the nanoholes. (**G**) Cross-section of the 3D nanoholes, randomly acquired on the nanoholes array (*n* = 3).

Three-dimensional nanostructures were fabricated on chitosan films via RP-AFL. Specifically, square nanoholes with lateral dimensions of 500 nm × 600 nm, 500 nm × 700 nm, and 500 nm × 800 nm were patterned on CH layers prepared from a 0.7% (*w*/*v*) polymer solution using RP-AFL (Figure 6A,B). The patterning process was performed in contact mode employing an NTEGRA AFM system equipped with NSG30 probes. A setpoint of 6 nA was applied and sequentially assigned from left to right and from top to bottom across the patterned area. All other processing parameters were maintained constant and equal to those used in the CP- and GP-AFL, including a scan direction from left to right, pulse duration τ = 300 ms, step of 10 nm, and a scan velocity of 0.25 μm/s.

Following the RP-AFL fabrication of the 15 nanostructures, the patterned area was characterized by high-resolution AFM imaging in semicontact mode by using NSG01 probes. The resulting topographical maps, processed with IA-P9 software, are presented in Figure 6C–F. The squared-shaped nanoholes exhibit well-resolved morphology with sharp edges. Anyway, the polymer carved from the nanostructures’ cavity accumulates along the perimeter of the structures, particularly at the lower edge, consistent with the imposed scanning direction of the AFM tip.

Topographical analysis was performed by extracting height profiles across the inner regions of the nanostructures (Figure 6G). The measured depth was (50 ± 2) nm, in good agreement with the thickness of the chitosan film. This value represents the mean and standard deviation calculated over all nanostructures, confirming that each feature extends down to the underlying SiO_x_ substrate. Notably, nanoholes patterned using a setpoint of 6.0 nA are approximately 30 nm deeper than nanogrooves patterned with the same setpoint by using the CP- and GP-AFL techniques. This increased depth can be attributed to the raster-mode patterning process, in which the AFM tip sculpts the sample surface along closely spaced lines in a template. As a result, repeated tip penetration in adjacent regions leads to a cumulative effect, ultimately producing deeper features. The regularity of the nanostructures indicates high patterning fidelity on the Ch layer and suggests that the AFM piezoelectric scanner operated without significant distortion, hysteresis, or other nonlinear artifacts requiring correction. As expected, increasing the applied normal force during patterning led to a degradation in structural quality, attributable to stronger tip–substrate interactions.

## 4. Conclusions

In this work, we demonstrated the feasibility of using chitosan thin films as a sustainable direct-write resist material for AFM-based nanolithography, achieving high-resolution patterning through Pulse–Atomic Force Lithography techniques. The fabricated films exhibited excellent uniformity, nanoscale roughness, and tunable thickness, confirming their suitability for nanofabrication applications. Through systematic force calibration via force spectroscopy, we established a direct correlation between the applied setpoint and the resulting nanostructure morphology. The CP-AFL approach enabled the fabrication of highly reproducible nanogrooves with tunable depth and width, while revealing direction-dependent anisotropies associated with probe geometry and tip–sample interaction mechanics. The GP-AFL technique further extended this capability, allowing the realization of graded-depth (2.5D) nanostructures with precise and predictable depth modulation. Finally, the RP-AFL method enabled the fabrication of well-defined three-dimensional nanoholes with depths corresponding to the full thickness of the chitosan layer, demonstrating complete material removal and high patterning fidelity over extended areas. Given that chitosan is readily soluble in dilute acetic acid, such patterned structures can be integrated into complex process flows where chitosan serves as a sacrificial protective layer, for instance, enabling metal deposition followed by lift-off. Importantly, the entire nanofabrication process was achieved without the use of conventional synthetic resists, toxic developers, or complex chemical modifications, thereby significantly reducing the environmental footprint of the process. These results highlight the strong potential of combining bio-derived materials with AFM-based nanolithography for the development of sustainable nanomanufacturing strategies. This work represents a proof-of-concept that incorporates environmentally friendly practices, including a biobased resist and a chemical-free lithographic technique, which are readily scalable and suitable for integration into eco-sustainable industrial manufacturing. Future work will focus on improving patterning throughput, exploring alternative biopolymer systems, and integrating these materials into functional devices, particularly in the fields of biodegradable electronics and biomedical applications.

## Figures and Tables

**Table 1:** Force values estimated by force spectroscopy.

Setpoint (nA)	0.5	1.0	1.5	2.0	2.5	3.0	3.5	4.0	4.5	5.0	5.5	6.0
Force (µN)	0.23	0.44	0.64	0.85	1.05	1.24	1.43	1.63	1.83	2.03	2.21	2.41
Err. (µN)	0.03	0.02	0.03	0.03	0.04	0.05	0.05	0.06	0.06	0.07	0.08	0.08

## Data Availability

The raw data supporting the conclusions of this article will be made available by the authors on request.
